# Frequency-comb enabled spectrum-correlation reflectometry for distributed fiber-optic sensing

**DOI:** 10.1038/s41377-025-02080-w

**Published:** 2026-01-01

**Authors:** Zhonghong Lin, Zhiyong Zhao, Huan He, Can Chen, Ming Tang, Marcelo A. Soto

**Affiliations:** 1 Wuhan National Lab for Optoelectronics (WNLO), School of Optical and Electronic Information, Huazhong University of Science and Technology, Wuhan, China; 2 Department of Electronics Engineering, Universidad Técnica Federico Santa María, Valparaíso, Chile

**Keywords:** Optical sensors, Imaging and sensing, Frequency combs

## Abstract

Distributed fiber-optic sensing has become an indispensable tool for large-scale structural and environmental monitoring, where spectral interrogation of backscattering light enables high-precision quantitative measurement of external perturbations. Conventional spectral analysis methods, typically based on frequency-domain serial interrogation or time-to-frequency mapping, face inherent trade-offs between measurement speed, dynamic strain measurement range, and system complexity. Here, we present a distributed frequency comb enabled spectrum-correlation reflectometry as a universal spectral analysis framework that leverages optical frequency comb for parallel multi-frequency interrogation, which is experimentally demonstrated in a phase-sensitive optical time-domain reflectometry (φ-OTDR) system. This method eliminates the need for large frequency scans, achieving more than tenfold improvement in measurement speed over the state-of-the-art spectral analysis methods. Compared to existing phase-demodulated φ-OTDR systems, this method enables vibration amplitude monitoring with a dynamic strain measurement range expanded by more than an order of magnitude, while intrinsically circumventing phase unwrapping issues and interference fading. This work establishes a new paradigm for distributed spectral analysis, providing a flexible and robust platform for a wide range of sensing technologies, including Rayleigh and Brillouin-based schemes, which may have significant impact for geophysics, seismology, civil engineering, and other fields.

## Abstract (web summary)

Frequency-comb enabled spectrum-correlation reflectometry employs a dual-sideband interleaved configuration to perform parallel multi-frequency interrogation, providing high frequency response over a broad optical spectral range.

## Graphical abstract

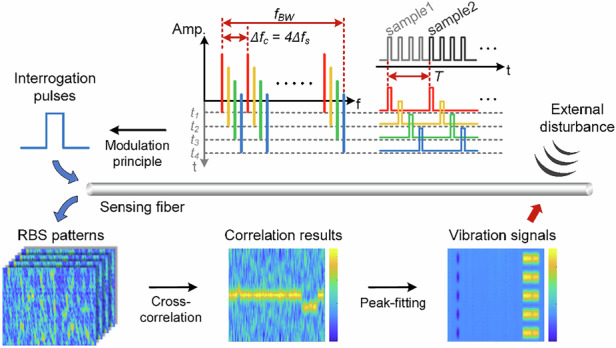

## Introduction

Distributed fiber-optic sensing is capable of continuously monitoring the spatially distributed evolution of environmental variables along a sensing fiber by exploiting light backscattering, which has been extensively used in geophysical exploration^1,2^, seismic surveillance^3,4^, and structure health monitoring^5,6^. Based on the frequency dependence of light backscattering within the fiber, accurate quantitative measurement of external perturbations is achieved through spectral analysis. For example, the frequency-scanned phase-sensitive optical time-domain reflectometry (φ-OTDR) schemes^7^ rely on the local frequency shift of the position-resolved Rayleigh backscattering (RBS) spectral patterns. Similarly, distributed Brillouin sensors extract the information of perturbations by measuring the frequency shift of the Brillouin gain spectrum^8^. From the spectral perspective, frequency domain interrogation plays a critical role in distributed fiber-optic sensing systems.

However, traditional frequency-scanned approaches^7^ reconstruct the optical spectrum by scanning the interrogation frequency sequentially, which is very time-consuming and severely limits the frequency response range. As a result, these methods are usually considered to be solutions that only allow for quasi-static perturbation monitoring^7^. Though dynamic measurement can be achieved by dramatically reducing the frequency switching times^9–12^, the stepwise scanning of the interrogation frequency inherently restricts the frequency response range, which remains an inevitable fundamental limitation of all frequency scanned schemes. An alternative strategy using chirped frequency modulation^13,14^ converts the frequency shift into the time domain, thus eliminates the need for frequency scanning and dramatically enhances the measurement speed. However, limited by the chirp range of the interrogation pulse, the measurable strain variation is usually small, typically in the sub-micro-strain scale^15^. Efforts to overcome this limitation, such as the pulse compression approach, require complex digital demodulation and suffer from laser phase noise and random polarization evolution, making practical implementation more challenging^15–18^.

To address these challenges, parallelization in the frequency domain emerges as a promising strategy. Known as a groundbreaking tool for precision metrology, the optical frequency comb (OFC) has found widespread applications in various fields, including astrophysics^19^, spectroscopy^20,21^ and optical telecommunications^22^. In particular, OFC enables parallel multi-frequency interrogation thanks to its evenly spaced and highly stable spectral lines across a broad spectral range, which makes it an ideal candidate for ultrafast, high-precision spectral analysis. Recently, the OFC has been introduced into Brillouin optical time domain analysis to achieve scanning-free Brillouin gain spectrum interrogation, thereby enabling dynamic measurement^23,24^. As for φ-OTDR systems, prior works have utilized the OFC for improving spatial resolution and signal-to-noise ratio (SNR) through dual-comb interferometry^25–27^. However, existing OFC-based φ-OTDR methods still rely on phase demodulation rather than spectral analysis, which introduces a slew-rate constraint^28^ and interference fading^29^, thus imposing a strict trade-off between the measurable strain range and the vibration frequency range.

In this work, we propose a fundamentally new framework for dynamic strain measurement with ultrahigh precision and large measurable strain range using OFC-based spectrum-correlation reflectometry (OFC-SCR). Different from the conventional time-domain or frequency-scanned methods, the OFC-SCR employs a broadband OFC to simultaneously interrogate the spectral patterns along the sensing fiber. Each comb-tooth samples a specific optical frequency within the spectrum, allowing the entire optical spectrum pattern to be captured, in principle, by a single-shot measurement. This way, the utilization of an OFC transforms sequential frequency scanning into a parallel frequency-domain process, enabling distributed dynamic measurement over a broadband optical spectral range. The proposed OFC-SCR method is theoretically analyzed and experimentally demonstrated in a φ-OTDR system, offering an order-of-magnitude improvement in measurement speed and dynamic strain range compared with the state-of-the-art φ-OTDR schemes, while inherently avoiding the limitations associated with phase demodulation and interference fading. The proposed framework establishes a scalable, robust, and high-resolution platform for real-time distributed vibration and strain analysis, with potential applications in geophysics, seismic surveillance, and structure health monitoring, among many other potential applications.

## Results

### Design principle of the digital OFC

**Fig. 1:**
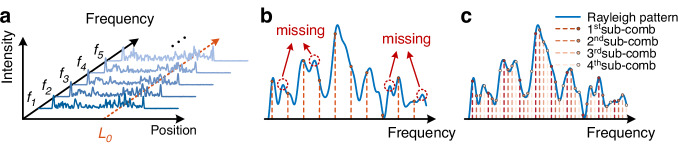
Principle of OFC-enabled φ-OTDR. **a** Illustration of the Rayleigh backscattered traces for different interrogation frequencies. **b** Rayleigh spectrum detected by a single OFC, where the blue curve denotes the Rayleigh spectrum at a given fiber position *L*_*0*_ and the red dashed lines denote the sampled values at corresponding frequencies. **c** Rayleigh spectrum detected by four equally spaced sub-combs, where the different sets of dots represent the sampled values of different sub-combs

Instead of scanning the optical frequency of a single-carrier optical pulse, as in the conventional frequency-scanned scheme^7^, the proposed OFC-SCR method modulates an OFC into the interrogation optical pulses. All the frequency components in the OFC must be mutually orthogonal, allowing them to be fully distinguished in the frequency domain. Each frequency component samples an individual RBS trace, with the frequency spacing of the OFC determining the spectral sampling interval. This permits the simultaneous interrogation of multiple frequency components of the RBS spectral response across a broad spectral range, as shown in Fig. 1a. This way, the frequency response of the system, so-called acoustic bandwidth of the sensor, is no longer directly determined by the number of interrogation frequencies, aside from the sensing fiber length, but instead depends on the number of sub-combs. Therefore, the proposed OFC-SCR method enables a significant improvement in the measurement speed compared to the traditional frequency-scanned scheme^7,9–12^.

As a spectral analysis method, the spectral sampling interval of the proposed method is a primary factor to be considered. According to the Nyquist sampling theorem, the spectral sampling interval $\Delta {f}_{s}$ must be smaller than half of the full-width-at-half-maximum of the Rayleigh spectral patterns^30^, which is equal to the inverse of the interrogation pulse width ${t}_{p}$; therefore, $\Delta {f}_{s}\le 1/2{t}_{p}$. However, the frequency spacing of the comb teeth $\Delta {f}_{c}$ must be larger than twice the full-width-at-half-maximum to fully distinguish all the frequency components. In addition, according to the Fourier transform theory, the period length of an OFC waveform is determined by the inverse of $\Delta {f}_{c}$ ^25^, which requires ${t}_{p}\ge 2/\Delta {f}_{c}$. The above two requirements impose the condition that $\Delta {f}_{c}\ge 4\Delta {f}_{s}$. Therefore, at least the use of four groups of sub-frequency-combs is required for each measurement to fully reconstruct the intact Rayleigh spectral patterns with no information loss, as shown in Fig. 1b, c. Employing less than four sub-combs violates the Nyquist sampling theorem, resulting in undersampled Rayleigh spectral patterns and reduced contrast between the main cross-correlation peak and its sidelobes, thereby increasing the probability of correlation peak-localization errors. On the contrary, increasing the number of sub-combs improves the frequency resolution and can mitigate the effects of noise in the cross-correlation spectrum, thereby enhancing the accuracy of the main correlation peak localization. However, this improvement comes at the expense of a reduced acoustic bandwidth in the sensing system.

Let us consider an OFC consisting of $m$ sets of sub-combs, equally spaced in the frequency domain, with a total of $n$ frequency components. The initial frequency component of the OFC is denoted as ${f}_{0}$, and the frequency spacing of the OFC is represented as $\Delta {f}_{s}$. Then, the frequency sequence of the entire OFC can be expressed as:

$$\left\{{f}_{0},{\,f}_{0}+\Delta {f}_{s},{\,f}_{0}+2\Delta {f}_{s},\cdots ,{f}_{0}+(n-1)\Delta {f}_{s}\right\}$$

Each of the $m$ sub-combs contains a subset of frequencies that are spaced by $\Delta {f}_{c}=m\Delta {f}_{s}$, which corresponds to the frequency spacing of the sub-combs. The frequency sequence of the $i$*-th* sub-comb is given by:

$$\left\{{f}_{i},{\,f}_{i}+m\Delta {f}_{s},{\,f}_{i}+2m\Delta {f}_{s},\cdots \right\}$$

where ${f}_{i}={f}_{0}+(i-1)\Delta {f}_{s}$, and $i=\mathrm{1,2,3},\cdots ,m$. Generally, the total number of frequency components $n$ is chosen to be an integer multiple of $m$, so that each sub-comb contains the same number of frequency components.

**Fig. 2:**
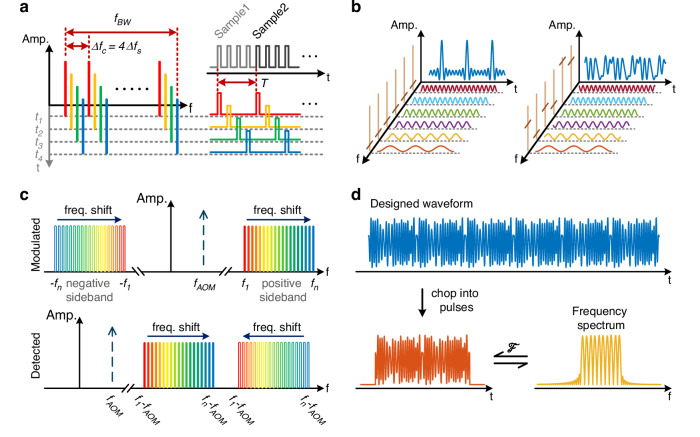
Modulation principle of the proposed OFC interrogation pulse. **a** Schematic diagram of the four sub-combs and their respective interrogation pulses, where different colors represent different sub-combs and the corresponding pulse sequence, so that four successive pulses constitute one valid φ-OTDR measurement. **b** Principle of PAPR reduction using random phase modulation, where the light brown lines along the frequency axis represent the frequency components, and the dark brown markers on them denote the initial phases of the corresponding frequency components. **c** Frequency characteristics of the modulated two sidebands in the optical domain and their detection in the electrical domain, where the negative sideband (marked by double lines) is spectrally inverted during coherent detection, while the positive sideband remains unchanged. **d** Temporal waveform exemplifying the time features of the signal composed of the designed OFC

To achieve the highest detectable vibration frequency, the number of sub-combs $m$ is chosen to be four in this work, which is the minimum possible number according to the previous analysis. These four sub-combs are interlaced with each other, as shown in Fig. 2a, with sequenced starting frequencies and identical frequency spacing. Four successive interrogation pulses, each carrying one of the sub-combs in order, are sequentially launched into the sensing fiber with a certain temporal interval determined by the pulse repetition period. The corresponding four successive φ-OTDR traces are processed as a single interrogation. Consequently, the entire interrogation procedure takes a period of $T$, as shown in Fig. 2a, which is four times the pulse repetition period, leading to a frequency response range that is one eight of the pulse repetition rate.

As shown in Fig. 2b, a signal carrying an OFC corresponds to a superposition of a series of sinusoidal functions in the time domain. If the spectral phase of the comb is uniform, the temporal waveform of the optical signal would appear as a train of short and transform-limited pulses with high peak power. This leads to a very high peak-to-average power ratio (PAPR) of the signal, which severely restricts the maximum optical power allowed in the sensing fiber that is determined by the onset of nonlinear effects^25^. To maximize the optical power launched into the sensing fiber, it is necessary to specifically design the spectral phase of the OFC to avoid the formation of high peak power pulses^25^. Random phase modulation is used in this work to reduce the PAPR. This way, the temporal trace of the random phase-modulated OFC signal becomes a periodic noise-like waveform with a much lower PAPR, and a period that equals the inverse of the frequency spacing, as shown in Fig. 2b. In particular, here the PAPR of the OFC is minimized using an exhaustive search over random phase distributions. This optimization is performed through Monte Carlo simulations, evaluating over one million phase combinations for each OFC. Specifically, for the OFC with a 400 MHz bandwidth, 10 MHz line spacing, and 40 spectral components, the PAPR is minimized to 6.5 dB. Compared to the unmodulated OFC waveform, which exhibits a PAPR of 13.0 dB, this represents a reduction of 6.5 dB. In the case of the OFC with 5 MHz line spacing and 80 spectral components, the PAPR is minimized to 5.5 dB, providing a 10.5 dB reduction compared to the unmodulated waveform, which exhibits a PAPR of 16.0 dB. Note however that even lower PAPR values may be achievable with different OFC configurations, particularly those employing a higher number of spectral lines. It is important to clarify that the proposed OFC-SCR method does not require precise phase matching between the probe OFC and the local oscillator (LO), unlike other schemes like the time-expanded φ-OTDR^25^. In our approach, all frequency components of the probe OFC beat coherently with a single LO, and frequency demultiplexing is used to separate the individual comb lines. In this context, the random initial phase of each comb line does not affect the frequency of the beat signals or the efficiency of the demultiplexing, provided that the frequency orthogonality of the comb is preserved. Although different initial phases modify the absolute Rayleigh spectral pattern, the method relies only on relative spectral shifts, not on the exact spectral shape. Therefore, sensing accuracy remains unaffected as long as the unperturbed spectral patterns are temporally stable, something primarily limited by the laser phase noise and environmental fluctuations.

In the proposed system, the OFC is generated through intensity modulation, which gives rise to two OFC sidebands around the optical carrier, as shown in Fig. 2c (upper subplot). When an external perturbation occurs, the frequency shift detected by the two OFCs moves in opposite directions because the OFC at optical frequencies lower than the carrier, here denoted as negative sideband, undergoes a spectral inversion during optical coherent detection^31^. To separate the two OFCs, an acousto-optic modulator (AOM) is used to generate a carrier frequency shift while pulse chopping, so that the two OFCs can be separated in the frequency domain by simple electrical or digital filtering. Assuming that the frequency shift induced by the AOM is ${f}_{{AOM}}$, and the first tooth of the designed OFC is ${f}_{1}$, then the starting frequencies of the received traces from the two OFCs are ${f}_{1}-{f}_{{AOM}}$ and ${f}_{1}+{f}_{{AOM}}$, respectively, as shown in Fig. 2c (lower subplot). Therefore, to avoid aliasing of the backscattering signals of the two OFCs, the bandwidth of the designed single OFC cannot exceed $2{f}_{{AOM}}$. Naturally, the available bandwidth of the frequency comb can be expanded to GHz scale by simply increasing the frequency shift between the signal light and the LO light. Figure 2d shows an example of the generated waveform of the designed OFC, which has periodic noise-like intensity distribution with a relatively low PAPR. Then, this waveform is chopped into pulses with a minimum pulse width ${t}_{p}=2/\Delta {f}_{c}$, securing that the linewidth of each frequency component matches the comb tooth spacing $\Delta {f}_{c}$, which provides the highest spatial resolution with the given OFC settings.

### Demodulation of the received signal

**Fig. 3:**
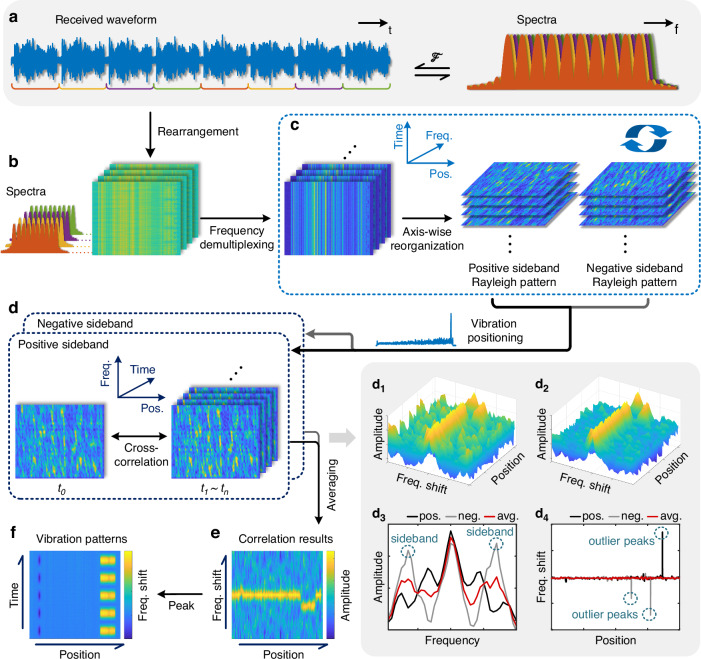
Demodulation principle of the proposed scheme. **a** Received RBS waveforms and corresponding spectrum, where the four colors represent the four sub-combs used in the proposed scheme. **b** Rearrangement of the received waveforms into four RBS matrices according to the fiber position, time, and the sequence of sub-combs. **c** Frequency demultiplexed RBS matrices and Rayleigh spectral patterns of the two OFC sidebands, representing the axis-wise reorganization of the three-dimensional data. **d** Cross-correlation principle of the Rayleigh spectral patterns of both OFC sidebands, with (d_1_) oblique top view of a correlation spectrum of a single OFC sideband. (d_2_) Oblique top view of the averaged correlation spectrum from both OFC sidebands. (d_3_) Comparison of the local correlation spectra obtained with the positive-frequency OFC, the negative-frequency OFC and the average of them at a specific fiber position, where high correlation sidebands can be observed. (d_4_) Peak-fitting results of the positive-frequency OFC, the negative-frequency OFC and the average of them along the sensing fiber, where several outliner peaks exist. **e** Averaged correlation spectrum of the two OFC sidebands. **f** Demodulated vibration pattern over time and distance along the fiber

The flow chart of the demodulation scheme used in this work is illustrated in Fig. 3. Let us consider the case of four sub-combs as an example, so each valid measurement is composed of the RBS signals obtained from four consecutive interrogation pulses, each one carrying one of the 4 sub-combs in sequence, as shown in Fig. 3a. The total number of interrogation frequencies is $n$, distributed across all the sub-combs. Therefore, each sub-comb contains $n/4$ frequencies, assuming $n$ is an integer multiple of $4$. The raw data is processed using digital coherent demodulation. For this, the original signal is firstly rearranged into a set of matrices according to the fiber position, time, and the sequence of sub-combs, as shown in Fig. 3b. As four sub-combs are used here, four matrices are obtained. Each of the four matrices carries one of the sub-combs, and is then multiplied by a set of sine waves and cosine waves according to the frequency components within the corresponding sub-comb. This process down-converts the signal to baseband, separating it into in-phase ($I$) and quadrature ($Q$) components. Then, both $I$ and $Q$ components are filtered in the frequency domain to remove noise. Finally, the filtered $I$ and $Q$ components are combined to reconstruct the signal amplitude for all interrogation frequencies, resulting in a series of frequency-demultiplexed RBS matrices. Each matrix contains the RBS traces measured at a specific interrogation frequency, arranged as a function of the fiber position and sampling time, as illustrated on the left side of Fig. 3c. To extract the Rayleigh spectral patterns, the three-dimensional dataset is read by fixing the sampling time. This axis-wise reorganization results in matrices containing the Rayleigh response as a function of the interrogation frequency and fiber position. In this way, these matrices provide the Rayleigh spectral patterns at each fiber location and are arranged in chronological order, as shown on the right side of Fig. 3c. External perturbations can be observed as shifts along the frequency axis within the obtained Rayleigh spectral patterns. Note that to obtain the RBS frequency shift induced by external perturbations at each fiber position, the Rayleigh spectral patterns obtained from both positive- and negative-frequency OFCs need to be independently processed as will be explained below, as their recorded frequency shifts are opposite.

Several alternative methods have been proposed in the literature to estimate the frequency shift of the Rayleigh spectral patterns^7,32–34^. Without loss of generality, the basic cross-correlation approach^7^ is employed in this work. The first demodulated RBS pattern of both positive- and negative-frequency OFCs is chosen as the reference pattern, and the cross-correlations are calculated between each Rayleigh spectral pattern sampled at ${t}_{1} \sim {t}_{n}$ and the reference pattern sampled at ${t}_{0}$, as shown in Fig. 3d. Since the frequency shifts of the Rayleigh spectral patterns from the positive- and negative-frequency OFCs are the same but with opposite shift directions, the correlation result from the negative-frequency OFC needs to be spectrally flipped and then averaged with the correlation result from the positive-frequency OFC. The average correlation spectrum is shown in Fig. 3e. The frequency shift at each fiber position is then obtained from the average correlation spectrum, which is linearly proportional to the external temperature and strain variations with typical sensitivities of ~1.33 GHz/K and ~152.44 MHz/με^35,36^, respectively. To retrieve external perturbations, quadratic curve fitting is used to estimate the spectral shift of the main correlation peak. This is achieved by performing peak fitting on spectral points that exceed a threshold level of 0.5 in the cross-correlation spectrum. The peak fitting results are then arranged in temporal order, yielding the external perturbation pattern as a function of time at each fiber location, as shown in Fig. 3f.

Note that the proposed processing requires the calculation of the cross-correlation of the two OFC spectral responses separately and then average the results. This way of processing is based on different considerations. First, the positive- and negative-frequency OFCs generate different Rayleigh spectral responses due to the distinct optical frequencies propagating through the fiber in each comb. Therefore, directly averaging the two spectral responses before correlation would change (distort) the local Rayleigh spectral shapes. While such averaging might slightly reduce noise, most noise suppression is already achieved through the cross-correlation itself, which acts as a low-pass filter. The most significant advantage lies in reducing unwanted cross-correlation sidelobes. Computing the cross-correlation spectra of the two sidebands separately yields two spectra with similar main correlation peak, indicating the actual frequency shifts caused by temperature or strain changes, but dissimilar sidelobe structures, due to the different Rayleigh spectral responses. Averaging these two local cross-correlation spectra effectively reduces the sidelobes and enhances the contrast of the main peak, reducing the probability of large errors^37^ in the correlation peak localization. For these reasons, averaging after the cross-correlation step proves more effective than averaging the spectral responses beforehand.

An example of the correlation of the Rayleigh spectral patterns obtained from a single OFC sideband is shown in Fig. 3d_1_, while the result of averaging the cross-correlation spectra from both OFC sidebands is shown in Fig. 3d_2_. The latter reveals a clearer main correlation peak, with reduced correlation sidebands. A comparison of the correlation spectra for the positive-frequency OFC, the negative-frequency OFC and the average of them at a specific fiber position with no external disturbance is shown in Fig. 3d_3__._ In this figure, clear correlation sidebands can be observed symmetrically around the true zero-frequency-shift peak when a single OFC sideband is used. These sidelobes are however highly suppressed in the average correlation spectrum. The frequency of the main correlation peak along the fiber is shown in Fig. 3d_4_, where several outlier peaks are present when using a single OFC sideband and no outlier peak exists in the averaged result. This comparison highlights the effectiveness of the averaging process in reducing large peak-fitting errors that are typically observed in conventional frequency-scanned φ-OTDR scheme^37^.

### Experimental results

To fully evaluate the performance of the proposed OFC-SCR method, several sets of experiments with different parameter settings have been conducted using two groups of OFC configurations. For all the experiments, the frequency difference between the probe and the LO introduced by the AOM is 200 MHz, resulting in an OFC of 400 MHz bandwidth. Note that the electrical frequency comb used for modulation ranges from 600 MHz to 1 GHz, so the received response of the upper-frequency OFC ranges from 400 MHz to 800 MHz in the detection electrical domain, while the response of the lower-frequency OFC ranges from 800 MHz to 1.2 GHz. Detailed experimental parameters of the two groups of OFC configurations are listed in Table 1, while the used experimental setup is described in the Methods section.

**Table 1:** The OFC configurations

Common parameters		
Frequency shift of AOM	200 MHz	
Measurement bandwidth	400 MHz	
Electrical comb band	600 MHz ~ 1 GHz	
**Different parameters**	**The 1**^**st**^ **set**	**The 2**^**nd**^ **set**
Pulse width	50 ns	100 ns
Frequency spacing ($\Delta {f}_{c}$)	40 MHz	20 MHz
Number of sub-combs	4	4
Frequency resolution	10 MHz	5 MHz
Number of frequencies	40	80

Firstly, the dynamic sensing capability of the proposed scheme is verified using the 1^st^ set of OFC configuration with a sensing fiber of 380 m and a pulse repetition rate of 200 kHz. Consequently, considering the use of four sub-combs, the effective sampling rate of the system is 50 kHz, resulting in a frequency response range (acoustic bandwidth) of 25 kHz. A piezo transducer (PZT) located at a distance of 365 m, with ~14 m fiber wound around it, is driven by a single sinusoidal signal of 0.25 V, whose frequency is set to be 6 kHz, 12 kHz and 24 kHz, respectively. It should be noted that the 24 kHz vibration signal is close to the maximum detectable vibration frequency of the sensor according to the Nyquist sampling theorem with the above parameter settings.

**Fig. 4:**
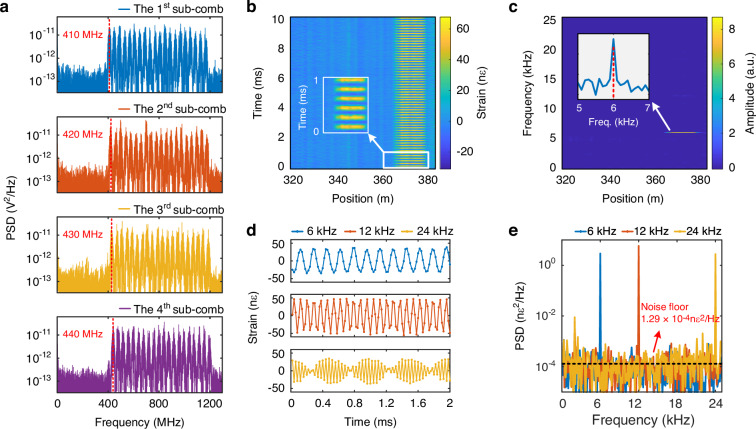
Experimental results for dynamic sensing using the 1^st^ set of OFC configuration. **a** PSD spectra of the received RBS traces, each carrying one of the four sub-combs. **b** Strain waterfall map between 320 m and 380 m, showing a vibration at 6 kHz, with an insert showing an enlarged view of the vibration over a 1 ms duration. **c** Spectrum as a function of the distance between 320 m and 380 m, with an insert showing the spectrum around 6 kHz at the fiber position of 370.2 m. **d** Set of three demodulated temporal waveforms at 370.2 m, corresponding to vibration frequencies of 6 kHz, 12 kHz and 24 kHz, respectively. **e** PSD spectra of the three vibrations measured at 370.2 m

The power spectral density (PSD) of the received RBS traces of the four successive sub-comb interrogation pulses are shown in Fig. 4a, demonstrating that all the interrogation frequency components can be clearly identified. As an example, the measured strain variation evolution of the 6 kHz vibration is shown in Fig. 4b with a color scale mapping the strain changes along the fiber, where the perturbation is clearly visible between 365 m and 379 m. The corresponding frequency spectrum as a function of fiber position is shown in Fig. 4c, where the peak at 6 kHz can be easily identified, indicating a good SNR of the vibration signal. In addition, the demodulated temporal waveforms of the three vibration signals are displayed in Fig. 4d. For a more detailed demonstration of the dynamic sensing capability, the detected time evolution of the cross-correlation spectrum is shown in the Supplementary Movies S1-S3 for the three vibration frequencies. On the other hand, Supplementary Movie S4 demonstrates the capability of the proposed method to detect larger strain variations, covering large part of the interrogated optical spectrum, at 0.5 kHz. Note that the envelope of the obtained 24 kHz vibration closely approximates a 1 kHz sinusoidal waveform, which corresponds to the difference frequency between the vibration signal and the Nyquist frequency. The PSDs of the vibration signals are plotted together in Fig. 4e, where all the three vibration frequency components can be clearly identified. The PSD noise floor, defined as the root mean square of the PSD excluding the strain signal and its second harmonic component induced by a nonlinear response of the PZT, is 1.29·10^-4^ nε^2^/Hz. This background noise corresponds to a strain resolution (sometimes called sensitivity) of ~11.4 pε/√Hz near the far fiber end. The experimental results conclusively demonstrate the ultra-high strain resolution of the proposed scheme in detecting high-frequency signals.

**Fig. 5:**
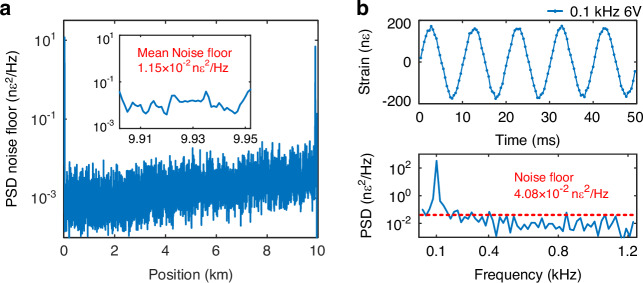
Experimental results obtained by the 2^nd^ set of OFC configuration with a 10 km sensing fiber. **a** PSD noise floor along the 10 km sensing fiber, with an insert showing an enlarged view of the fiber section between the PZT and the far end of the fiber. **b** Demodulated temporal waveform and corresponding PSD of a vibration of 0.1 kHz applied at 9893.6 m

In order to evaluate the long-range sensing performance of the proposed scheme, a 10 km sensing fiber is used with the 2^nd^ set of the OFC configuration. Note that for this longer fiber, nonlinear effects become more significant than in the previous case using a much shorter sensing fiber. By varying the peak power of the OFC waveform, we experimentally estimate the nonlinear threshold to be approximately 27.5 dBm. To operate below this limit, the input peak pulse power used in the experiment is set to 22.1 dBm, corresponding to 6.1 dBm per spectral line. A PZT, with ~10 m fiber wound around it, is placed at a distance of 9890 m of the sensing fiber and is driven by a sinusoidal signal with a frequency of 0.1 kHz and an amplitude of 6 V. The local PSD noise floor along the sensing fiber is shown in Fig. 5a, with a mean noise floor of 1.15·10^-2^ nε^2^/Hz at the end of the 10 km sensing fiber, corresponding to a mean strain resolution of ~107.2 pε/√Hz. The demodulated temporal waveform and the PSD spectrum of the vibration signal at 9893.6 m are illustrated in Fig. 5b, showing a local noise floor of 4.08·10^-2^ nε^2^/Hz, which corresponds to a local strain resolution of ~201.9 pε/√Hz. These experimental results verify the capability of the proposed scheme to accurately measure mechanical vibrations along a 10 km sensing distance.

Finally, the proposed scheme is thoroughly compared with the conventional frequency-scanned method^10–12^ and the phase-demodulated φ-OTDR scheme based on heterodyne optical detection^38^. The length of the used sensing fiber is 2.4 km, and a 400 Hz/3 V vibration perturbation is generated using a PZT at approximately 2340 m. The first OFC configuration is used for the experiments, with a pulse interval of 25 μs, corresponding to a pulse repetition rate of 40 kHz. Since four sub-combs are used to form a single measurement, the effective sampling rate of the system is 10 kHz. Each sub-comb contains 10 interrogation frequencies with a frequency interval of 40 MHz, resulting in a total of 40 frequencies. For fair comparison, the frequency-scanned method is configured with the same sweep interval and number of frequencies, resulting in an effective sampling rate of 1 kHz. The system setup of the frequency-scanned φ-OTDR experiment is similar to the well-known conventional scheme^7,12^, and differs from the one in Fig. 7 in the pulse modulation and optical detection. In particular, a semiconductor optical amplifier is used for pulse modulation in the frequency-scanned experiment instead of the AOM used in Fig. 7. In addition, the RBS signal is obtained with direct detection rather than with the coherent detection used in Fig. 7. For fair comparison, the peak power of the interrogation pulses is set approximately equal in all schemes.

**Fig. 6:**
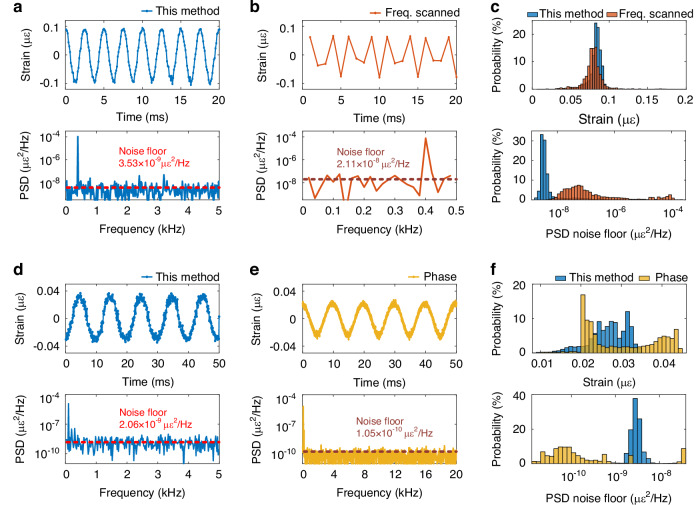
Comparison of the proposed method with the conventional frequency-scanned method and the phase-demodulated φ-OTDR method. Demodulated waveform and the corresponding PSD spectrum of a 400 Hz vibration with 3 V driving voltage applied at 2350.8 m for **a** the OFC-SCR method using the 1^st^ set of OFC configuration, and **b** the conventional frequency-scanned method. **c** Probability distribution of the demodulated strain amplitude and PSD noise floor for the two methods. Demodulated waveform and the corresponding PSD spectrum of a 100 Hz vibration with 1 V driving voltage applied at 2350.8 m for **d** the OFC-SCR method using the 1^st^ set of OFC configuration, and **e** the implemented phase-demodulated φ-OTDR scheme. **f** Probability distribution of the demodulated strain amplitude and PSD noise floor for the two methods

The demodulated strain waveforms and the corresponding PSD detected by these two methods are shown in Fig. 6a, b, respectively. Although the total sampling duration for both schemes is 50 ms, only 20 ms segments of the time-domain waveforms are shown for clarity. Additionally, ten independent measurements are performed for each method. While both detection schemes could differ in the noise levels, the observed difference in noise floor is mainly due to the differences in the sampling rate of the two schemes, which cause noise aliasing in the classical frequency-scanned φ-OTDR scheme. This means that noise frequency components over 500 Hz are folded into the 0–500 Hz spectral density range shown in Fig. 6b, explaining the larger noise power spectral density in this configuration compared to Fig. 6a. On the other hand, given that the vibration length spans 14 m, only the central fiber section of 9 m-long within the perturbed section is selected for further analysis of the retrieved dynamic strain, removing the estimation deviations introduced by the rising and falling edges determined by the spatial resolution. The probability distributions of the demodulated strain amplitude and the PSD noise floor of the two methods, for all sampling points within the central section, are presented in Fig. 6c. The experimental results indicate that both the proposed scheme and the traditional frequency-scanned method can successfully detect vibrations with comparable standard deviations, as shown in Fig. 6c (upper subplot). However, the distribution of PSD noise floor of the proposed scheme exhibits a lower mean value and a smaller standard deviation, as shown in Fig. 6c (lower subplot), which demonstrates an improved sensing sensitivity and enhanced robustness compared to the traditional frequency-scanned method.

**Fig. 7:**
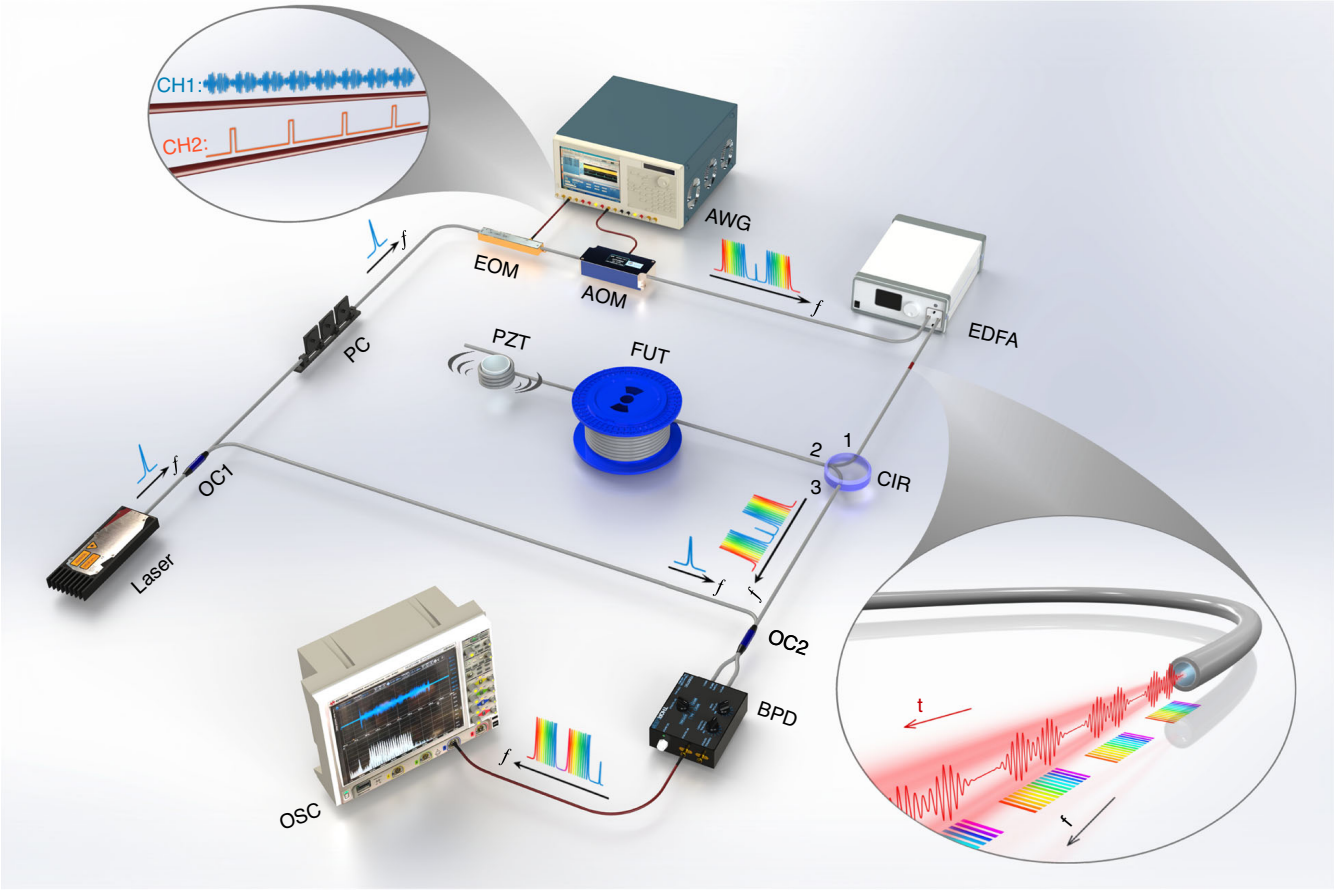
Experimental setup. PC: polarization controller; EOM: electro-optic modulator; AOM: acousto-optic modulator; AWG: arbitrary waveform generator; EDFA: Erbium-doped fiber amplifier; FUT: fiber-under-test; PZT: piezo transducer; BPD: balanced photodetector; OSC: oscilloscope; OC1: 90:10 optical coupler; OC2: 50:50 optical coupler; CIR: circulator. The AWG channel 1 is connected to the EOM, and the AWG channel 2 is connected to the AOM

The comparison of the proposed scheme and the phase-demodulated φ-OTDR scheme has also been performed with the PZT driven by a 100 Hz sine wave of 1 V amplitude. The experimental setup implemented for the phase-demodulated scheme is similar to the optical heterodyne detection scheme shown in Fig. 7, except that it excludes the OFC and the EOM for double-sideband modulation. Compared to the previous case, here a smaller vibration signal is generated, as the measurable strain range of the phase-demodulated φ-OTDR scheme is highly limited. Similar to the previous case, ten independent measurements are performed, selecting for analysis only the central fiber section of 9 m within the 14 m vibration region. The detected vibrations with the two schemes are presented in Fig. 6d and Fig. 6e, while the probability distributions of the demodulated strain amplitude and the PSD noise floor of the two methods are presented in Fig. 6f. The result reveals that although a lower mean value of the PSD noise floor is achieved for most measurements, the amplitude of the demodulated strain is highly scattered owing the presence of phase noise and Rayleigh intensity fading, which indicates less reliable detection results. In contrast, the proposed method shows superior stability and reliability, as evidenced by the concentrated distributions of the demodulated strain amplitude and PSD noise floor. Besides, it must be considered that the phase unwrapping procedure in the phase demodulated scheme imposes a strict requirement for the slew-rate^28^, which requires that the phase change between two consecutive measurements remains below π. This limitation restricts the maximum detectable strain amplitude of high frequency vibrations, especially when the frequency approaches the Nyquist frequency of the system. It must be pointed out that, in the implemented phase demodulated φ-OTDR scheme, the maximum detectable strain amplitude is limited to only 0.07 με for signals at the Nyquist frequency, assuming a π phase change between two consecutive measurements with a 5 m spatial resolution^39^, which turns out to be much smaller than that provided by the proposed scheme.

## Discussion

A novel dynamic strain sensing scheme with sub-nano strain resolution based on the OFC-SCR configuration has been proposed and demonstrated in this work. As the OFC consists of many optical frequency components, the proposed system can capture the Rayleigh spectral pattern in a single-shot measurement (or with a reduced number of acquisitions), eliminating the need for the time-consuming frequency scanning procedure during spectral analysis. This feature allows for more than tenfold improvement in frequency response range compared to the frequency-scanned scheme, without sacrificing its measurand resolution. Compared to the traditional frequency-scanned φ-OTDR^7,9–12^, the maximum detectable frequency of the proposed scheme is independent of the number of interrogation frequencies, being only dependent on the number of the used sub-combs and the length of the sensing fiber. In addition, the proposed scheme provides a much larger measurable strain variation range compared to the traditional phase-demodulation-based schemes with no slew-rate limitations.

The sensing performance of the proposed OFC-SCR method reflects a careful balance between several interacting system variables. A key limitation of the proposed scheme is the SNR of each interrogation frequency in the OFC. To avoid nonlinear effects, like Kerr effects, the peak power of each frequency component in the comb is naturally lower than that of the single-carrier pulse in the reference schemes. This power reduction constrains the SNR of the obtained spectral patterns, inherently limiting the sensing distance. As a result, the sensing distance of the proposed method is not comparable to traditional long-range sensing schemes. However, the sensing distance of the proposed method can potentially be extended by employing many of the existing methods for SNR enhancement, such as the distributed or lumped optical amplification^40–42^, optical pulse coding^43–45^ or using Rayleigh-enhanced fibers^12,16^.

The overall bandwidth of the OFC is another key parameter that influences the system performance. Increasing the OFC bandwidth while keeping a fixed spectral sampling interval allows the Rayleigh spectral pattern to be measured over a broader range, thereby extending the measurable range of temperature or strain variations. Additionally, the larger number of spectral samples enhances the contrast between the main peak and sidelobes in the correlation spectrum, improving the accuracy of the correlation peak localization, and, consequently, the measurand resolution. However, increasing the number of frequency components also spreads the available optical power across more frequencies, reducing the peak power per line. This degrades the SNR of the Rayleigh traces measured by each spectral line, introducing a trade-off between the maximum measurable strain or temperature and measurand resolution. In the current proof-of-concept setup, the OFC bandwidth is limited by the AOM due to the double-sideband modulation, which gives a typical measurable strain range of several microstrains. Switching to a single-sideband modulation could remove this bandwidth constraint, allowing for a broader strain range and a higher number of interrogation frequencies. However, this also increases the risk of large errors in peak identification, particularly under noisy conditions. Therefore, optimizing the system configuration and tuning the key parameters of the proposed method requires a careful balance between strain range, measurement resolution, SNR, and robustness against correlation peak-localization errors.

The spatial resolution of the proposed OFC-SCR method is determined by the temporal width of the probe pulse. Shorter pulses offer higher spatial resolutions, but broaden the Rayleigh spectral pattern, reducing the number of comb lines allowed in each sub-comb, and consequently, reducing the frequency resolution and spectral correlation accuracy. In this proof-of-concept implementation, moderate spatial resolutions (5 to 10 m) are employed to ensure sufficient spectral sampling density and robust correlation performance across the full fiber length. These resolutions are still comparable to those of commercial distributed acoustic sensors, typically ranging from 5 to 20 m. This choice is also based on the available OFC modulation bandwidth (400 MHz) and target dynamic sensing performance. Nevertheless, higher spatial resolutions are completely achievable with reliable performance, especially when the induced spectral shifts are relatively small. Further enhancements of the spatial resolution without compromising sensing accuracy and demodulation reliability remains an important goal for future work.

Similar to the traditional frequency-scanned scheme, the proposed OFC-SCR method needs to quantify the strain or temperature changes by calculating the frequency-shift of the Rayleigh spectral patterns. This is obtained by calculating the cross-correlation between the local measured Rayleigh spectral response with a local spectral reference. In this proof-of-concept, processing 800 consecutive measurements of 20 ms signals with 100 M samples along the fiber requires 4 minutes on a server equipped with an Intel Xeon Gold 6136 CPU @ 3.00 GHz and 256 GB RAM. Note that, in our current implementation, the frequency-demultiplexing and cross-correlation are calculated entirely on a computer processing unit (CPU) in a serial, off-line manner, resulting in the main contributors to the overall processing time. However, both operations involve large-scale array manipulations, including fast Fourier transform (FFT) and inverse FFT (IFFT) calculations, which are well suited for parallel processing. Substantial speed improvements can be achieved using parallelized processing strategies, such as graphic processing unit (GPU) acceleration^46^ or FPGA-based pipelined processing^47^. Specifically, the time complexity of FFT-based demultiplexing is *O(MNlogN)*, where *M* is the number of interrogation frequencies and *N* is the number the sampling points per frequency^48^. On the other hand, the time complexity of the cross-correlation is *O(M’N’*^*2*^*)*, where *M’* is the number of correlation frames and *N’* is the number of samples per frame^49^. By parallelizing the processing of different frequencies or frames, the demodulation speed can be improved by approximately two orders of magnitude^46,47^. In addition, alternative spectral matching methods that permit lower computational complexity and faster processing than the classical cross-correlation could be used, such as the least mean square algorithm^32^, local similarity matching^33^, and the phase cross-correlation^34^. In addition, the cross correlation itself could be efficiently computed using FFT on a GPU^50^, offering the potential for substantial reduction in processing time. With these optimizations, the current 4-minute processing time could be reduced to sub-second levels, offering strong potential for real-time operation. Overall, the proposed OFC-SCR approach offers a flexible and cost-effective solution for sensing scenarios requiring simultaneously high accuracy and large frequency response range.

## Materials and methods

### Experimental setup

The experimental setup used for the proposed scheme is shown in Fig. 7. A continuous-wave light from a coherent laser operating at 1550.12 nm with linewidth of ~1 kHz is split into two parts via a 90:10 coupler: 90% is used as the probe light and 10% serves as the local oscillator (LO). The probe light is first modulated by an electro-optic modulator (EOM) to generate frequency-comb signal using an arbitrary waveform generator (AWG) with carrier-suppressed double sideband modulation. The modulated probe light is then chopped into pulses by a 200 MHz acousto-optic modulator (AOM). After being amplified by an Erbium-doped fiber amplifier (EDFA), the probe light passes through a narrowband FBG to filter out the amplified spontaneous emission noise that is introduced by the EDFA, and then it is injected into the fiber-under-test (FUT) through a circulator. A piezoelectric transducer (PZT) that is driven by an arbitrary function generator (not shown in the setup) is used to generate mechanical vibrations to the sensing fiber. At the receiver side, the RBS signal is mixed with the LO in a 3 dB coupler, and the beating signal is then detected by a balanced photodetector (BPD) with 1.6 GHz bandwidth and acquired by a digital sampling oscilloscope. Finally, the measurements are processed on a computer.

## Supplementary information

Supplementary Information for Frequency-comb enabled spectrum-correlation reflectometry for distributed fiber-optic sensing

Supplementary Movies

## Data Availability

All data files can be obtained from the corresponding authors upon reasonable request.
