# Supplementary material for: Frequency-comb enabled spectrum-correlation reflectometry for distributed fiber-optic sensing
Source: Light Sci Appl. 2026 Jan 1;15:11. doi: 10.1038/s41377-025-02080-w (PMC12756320; doi:10.1038/s41377-025-02080-w)
Supplement: Supplementary file 1 — Supplementary Information for Frequency-comb enabled spectrum-correlation reflectometry for distributed fiber-optic sensing [file 41377_2025_2080_MOESM1_ESM.docx]

Supplementary Information for

**Frequency-comb enabled spectrum-correlation reflectometry for distributed fiber-optic sensing**

Zhonghong Lin^1^, Zhiyong Zhao^1,^ ^*^, Huan He^1^, Can Chen^1^, Ming Tang^1, *^, and Marcelo A. Soto^2^

^1^Wuhan National Lab for Optoelectronics (WNLO), School of Optical and Electronic Information, Huazhong University of Science and Technology, Wuhan, 430074, China

^2^Department of Electronics Engineering, Universidad Técnica Federico Santa María, 2390123 Valparaíso, Chile.

Email: *zhiyongzhao@hust.edu.cn; tangming@mail.hust.edu.cn*

***Supplementary Movie S1***

Detected time evolution of the cross-correlation spectrum along the sensing fiber with a vibration signal at 6 kHz between 365 m and 379 m of the fiber.

***Supplementary Movie S2***

Detected time evolution of the cross-correlation spectrum along the sensing fiber with a vibration signal at 12 kHz between 365 m and 379 m of the fiber.

***Supplementary Movie S3***

Detected time evolution of the cross-correlation spectrum along the sensing fiber with a vibration signal at 24 kHz between 365 m and 379 m of the fiber.

***Supplementary Movie S4***

Detected time evolution of the cross-correlation spectrum along the sensing fiber with a larger-amplitude vibration signal at 0.5 kHz between 460 m and 474 m of the fiber.
